# Measuring Implicit Attitudes Toward Digital Health Technologies in Older Adults Using an Influence-Aware Affect Misattribution Procedure: Development and Feasibility Study

**DOI:** 10.2196/87989

**Published:** 2026-08-18

**Authors:** Anna Vinnikova, Jinyue Zhan, Kailing Jin, Xianfeng Ding, Wei Sang, Weina Xu, Qian Yang

**Affiliations:** 1 Department of General Medicine and School of Public Health, The Fourth Affiliated Hospital, and International School of Medicine, Zhejiang University School of Medicine, Zhejiang University, 866 Yuhangtang Rd, Hangzhou, Zhejiang, 310058, China, 86 1875818112; 2 Zhejiang Key Laboratory of Intelligent Preventive Medicine, Zhejiang University, Hangzhou, Zhejiang, China; 3 Department of Geriatrics, School of Public Health, The Fourth Affiliated Hospital, and International School of Medicine, Zhejiang University School of Medicine, Zhejiang University, Hangzhou, Zhejiang, China; 4 Infection Control Department, Lishui City People's Hospital, Lishui, Zhejiang, China; 5 Department of Infection Management and Preventive Health Care, Zhejiang University Affiliated Zhejiang Hospital, Hangzhou, Zhejiang, China; 6 School of Psychology, Central China Normal University, Wuhan, Hubei, China; 7 Faculty of Health and Wellness, City University of Macau, Macau SAR, Macau SAR, China; 8 Department of Geriatric Center for Regeneration and Aging Medicine, The Fourth Affiliated Hospital of School of Medicine, and International School of Medicine, International Institutes of Medicine, Zhejiang University, Yiwu, Zhejiang, China

**Keywords:** older adults, digital health, technology adoption, implicit attitudes, affect misattribution procedure, usability, behavioral science

## Abstract

**Background:**

Older adults often express positive attitudes toward digital health technologies in surveys, yet adoption remains low. Self-report measures may not capture automatic affective reactions such as anxiety or distrust. Implicit paradigms such as the affect misattribution procedure (AMP) can reveal these automatic attitudes, but parameters optimized for younger adults may not be suitable for older adults because of age-related slowing and changes in visual processing.

**Objective:**

This study aimed to adapt and evaluate an influence-aware affect misattribution procedure (IA-AMP) for measuring implicit attitudes of older adults toward digital health technologies and to identify an age-appropriate prime duration that balances affect transfer strength with minimal conscious awareness.

**Methods:**

A 2-phase methodological adaptation and feasibility study was conducted among older adults (aged ≥60 y). Phase 1 (n=40) involved the development and validation of age-relevant synthetic images depicting older adults using digital health tools. The images were evaluated based on valence, arousal, thematic relevance, and low-level perceptual features. Phase 2 (n=56) implemented an IA-AMP with 3 prime durations (75, 350, and 425 ms) across 2 sequential cohorts. The first cohort (batch 2A, n=29) used the initial awareness probe, whereas the second cohort (batch 2B, n=27) used a simplified awareness interface. The core IA-AMP target judgment task remained unchanged across batches. The primary inferential outcome was the binary trial-level target judgment, coded as pleasant or unpleasant. Trial-level responses were analyzed using binomial logistic mixed-effects models with prime valence, prime duration, their interaction, and batch as fixed effects, along with random intercepts for participant and prime image. Awareness analyses were restricted to batch 2B.

**Results:**

The primary generalized linear mixed-effects model showed a significant prime valence × prime duration interaction (*χ*^2^_2_=38.1; *P*<.001). Positive primes increased the odds of pleasant target judgments relative to negative primes at all durations (odds ratio [OR] 11.2, 95% CI 5.8-21.6, at 75 ms; OR 31.9, 95% CI 16.0-63.4, at 350 ms; and OR 45.6, 95% CI 22.1-94.0, at 425 ms; all Holm-adjusted *P*<.001). The positive-negative contrast was smaller at 75 milliseconds than at 350 and 425 milliseconds, whereas the contrasts at 350 and 425 milliseconds did not differ significantly. Batch sensitivity analyses showed stronger overall priming in batch 2B, but the prime valence × prime duration × batch interaction was not significant (*χ*^2^_2_=0.76; *P*=.68).

**Conclusions:**

The IA-AMP offers a promising approach for assessing the affective responses of older adults to digital health technologies beyond self-report. A prime duration of approximately 350 milliseconds appears to be a practical calibration point for AMP studies involving older adults, producing strong affect transfer effects while avoiding the longest exposure duration. Because reported influence awareness was common, AMP effects should be interpreted alongside awareness measures rather than as awareness-free implicit attitudes. AMP-based affective measures may complement usability and adoption research by identifying emotional responses that users may not readily articulate, thereby supporting more inclusive and evidence-informed development, evaluation, and implementation of digital health technologies for aging populations.

## Introduction

### Background

As global populations age, maintaining health, independence, and quality of life for older adults has become a public health priority. Digital health technologies, such as telemedicine platforms, wearable sensors, and smart home monitoring systems, are increasingly used to support older adults in managing chronic conditions and maintaining independence while aging in place. These tools can enhance health outcomes, reduce hospitalizations, and promote independent living by extending clinical monitoring into the home environment and supporting self-management [1,2]. However, despite these benefits, adoption remains limited. Many older adults report anxiety, low confidence, and mistrust toward digital tools, reducing engagement even when access and technical support are available [3]. Understanding both conscious and automatic emotional reactions to these technologies is crucial because affective comfort and trust often predict sustained use and adherence to digital health programs.

Most research on technology adoption of older adults relies on self-report measures. However, older adults may provide overly positive responses due to social desirability or limited self-awareness of their emotional responses. As a result, surveys may underestimate discomfort or resistance toward digital health tools [4]. Complementary assessment approaches are therefore needed to uncover emotional barriers that older adults may not express directly. Implicit measures offer one such approach by indexing automatic affective responses that operate outside conscious awareness and sometimes predict behavior beyond explicit reports [5,6]. By capturing intuitive reactions rather than deliberate opinions, these measures can reveal how well a technology truly aligns with users’ needs and sense of security.

### Implicit Attitudes and Measurement Gaps

Implicit attitudes, defined as automatic positive or negative reactions that occur outside conscious awareness [5], can shape real-world behavior, including technology use. Measuring these implicit responses can reveal how older adults actually feel about digital health technologies, even when they cannot or do not verbalize these attitudes. The affect misattribution procedure (AMP) is a validated tool for capturing such automatic affect transfer [7]. In this task, participants rate neutral stimuli that are briefly preceded by an affective “prime” (eg, an image of a health device), and the prime’s emotional valence tends to bias evaluations of the neutral target. This paradigm has been used to study implicit evaluations of political and social stimuli [8,9], but its application to digital health contexts and older populations remains limited.

The AMP has been widely used in studies involving younger adults [7] but has rarely been tested in older populations or with age-relevant digital health imagery. Age-related differences in processing speed and attention suggest that standard AMP settings (approximately 75-ms primes) may be suboptimal for older adults, as slightly longer exposures may improve perceptual registration while also increasing the likelihood of conscious awareness [10,11]. Moreover, ecological validity matters in later life. Older adults may process age-congruent faces differently from younger faces, which highlights the importance of age-relevant materials when assessing affect [12]. Therefore, using stimuli that depict older adults in familiar health technology scenarios may produce more meaningful and representative affective responses.

### Current Knowledge Gap

Prior work also indicates that awareness of primes can modulate AMP outcomes, although the direction and interpretation of this modulation remain debated [7]. Some findings suggest that conscious awareness amplifies affect transfer [13], whereas others view awareness reports as post hoc interpretations of implicit responses [14]. For aging research, the practical challenge is to measure automatic affective responses to digital health cues while minimizing demand characteristics and undue reliance on self-reported awareness [7]. To address this gap, there is a need to adapt implicit attitude paradigms so that they remain both sensitive and truly implicit when used with older adults. Determining timing parameters that maximize affect transfer while minimizing awareness may help clarify how best to assess older adults’ spontaneous emotional reactions to digital health technologies.

### Objective

This study tested whether the AMP can sensitively detect older adults’ implicit attitudes toward digital health technologies and examined task parameters that optimize performance for this population. We conducted a 2-phase study with older adults recruited from a hospital setting to develop and validate age-relevant prime images depicting realistic digital health scenarios to ensure ecological validity and administer an AMP with 3 prime durations (75, 350, and 425 ms) and trial-level awareness assessments to evaluate whether affect transfer occurs, whether its magnitude varies by duration, and whether AMP effects persist on trials without reported awareness.

Our goal was to provide an age-appropriate, scalable, and health-relevant method to uncover unspoken comfort or hesitation toward digital health tools so that designers and clinicians can better tailor user-centered interventions that support trust and engagement. By identifying optimal timing parameters for implicit attitude assessment in older adults, this work also contributes a new methodological resource for evaluating emotional readiness and acceptance of digital health technologies.

## Methods

### Study Design and Overview

**Figure 1.**
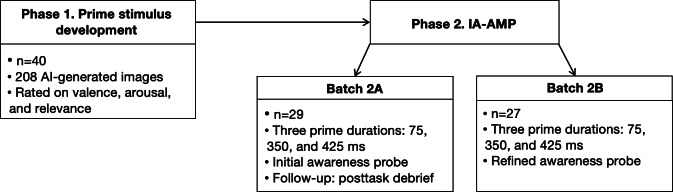
The overall study flow. IA-AMP: influence-aware affect misattribution procedure.

We conducted a 2-phase methodological adaptation and feasibility study with older adults to examine whether AMP can capture implicit attitudes toward digital health technologies and to identify timing parameters appropriate for older participants. Phase 1 involved development and validation of age-relevant prime images depicting older adults in digital health contexts, evaluating perceptual balance and affective clarity. Phase 2 was conducted across 2 sequential cohorts: batch 2A and batch 2B. Both cohorts completed the same core influence-aware affect misattribution procedure (IA-AMP) target judgment task, in which participants evaluated ambiguous target images as pleasant or unpleasant after positive or negative digital health primes or gray-square baseline trials. Batch 2A used the initial awareness-probe interface. Because no awareness responses were recorded in batch 2A despite explicit instructions, participant debriefings were used to refine the awareness-probe interface for batch 2B. The batch 2B procedure simplified the awareness response but retained the same core IA-AMP target judgment task. Therefore, both cohorts were included in the primary AMP analysis, whereas awareness-related inference was restricted to batch 2B. All procedures were conducted in Mandarin. Figure 1 illustrates the overall study flow.

### Ethical Considerations

Both studies were approved by the Ethics Committee of the School of Public Health, Zhejiang University (approval ID ZGL202411-1). All participants provided written informed consent before participation. Prime stimuli were synthetically generated to avoid any identifiable human participants; no personal data were collected or stored.

### Setting, Eligibility, and Recruitment

Participants aged ≥60 years were recruited from an inpatient geriatric department of a hospital in Hangzhou, China. Testing was conducted individually in a quiet, well-lit room using a standard desktop computer with a full keyboard. Inclusion criteria included normal or corrected-to-normal vision and the ability to understand and perform computerized tasks. Exclusion criteria included visual impairment, a history of neurological or psychiatric conditions (eg, stroke, dementia, Parkinson disease, and major depression), use of medications with cognitive side effects, motor impairments preventing keyboard use, or noncompliance during practice (eg, pressing the same response key on all trials).

### Phase 1: Stimulus Development and Validation

#### Objective

The first phase aimed to create a perceptually balanced, age-relevant set of positive and negative digital health primes and verify affective clarity and thematic relevance for older adults.

#### Stimulus Generation and Screening

A pool of 208 AI-generated images depicting older adults engaging with digital health technologies was created using Imagen 3 (Google). Prompts systematically varied 3 content dimensions: older-adult descriptors (eg, “senior” and “retired individual”), digital health contexts (eg, telemedicine, wearable sensors, and health apps), and emotional expressions (eg, happy, satisfied, frustrated, concerned, and neutral). All images were synthetic and were pilot-screened by 3 independent researchers for realism, clarity of emotional expression, cultural appropriateness, and absence of identifiable or sensitive content before inclusion in the task.

#### Perceptual Control and Preselection

To minimize low-level visual confounds, all images were processed in Python (OpenCV) to extract spatial frequency (2D Fourier transform on grayscale), chromatic complexity (color entropy in HSV), and luminance (mean intensity of 0‐255). Feature values were *z*-standardized, and Euclidean distance from the global centroid (0, 0, 0) was calculated to quantify perceptual deviation. We retained 25 images per valence category (positive, neutral, and negative; N=75) with the smallest distances for the behavioral rating phase.

#### Participants

A total of 40 older adults completed the rating task (2 additional participants were excluded due to noncompliance). Demographic information is summarized in Table 1.

**Table 1.** Demographic characteristics (study 1, n=40).

Variable	Value
Age (y), mean (SD)	68 (6.25)
Gender: female, n (%)	19 (47.5)
Education, n (%)	
Primary school	10 (25)
Middle school	14 (35)
High school	9 (22.5)
Junior college	4 (10)
Undergraduate	3 (7.5)
Residence, n (%)	
Urban	27 (67.5)
Rural	13 (32.5)
DHT^a^ users, n (%)	30 (75)

a DHT: digital health technology.

#### Procedure

After 10 practice trials, each participant viewed the 75 preselected images once in randomized order (500 × 400 pixels). To prevent cross-dimensional interference, participants were randomly assigned to rate either valence or arousal (7-point scale); all participants also rated relevance (7-point scale) to the theme “older adults using digital health.” Standardized instructions in Mandarin were presented onscreen and reiterated verbally. Responses were recorded by mouse click, and the task advanced automatically to the next trial.

#### Outcome Measures and Data Analysis

For each image, we computed mean ratings of valence, arousal, and relevance. Interrater reliability was estimated using the intraclass correlation coefficient (ICC[2,k]). Images were retained if they met predefined thresholds: valence≥5 (positive), ≤3 (negative), or 3.5 to 4.5 (neutral); relevance≥4; SD<1.5 for valence and relevance; and ICC≥0.70. Because neutral human images were not consistently rated as neutral, the neutral condition for the AMP task in phase 2 was operationalized as uniform gray squares, serving as a perceptual baseline. Normality was assessed using Shapiro-Wilk tests; group comparisons on perceptual features used ANOVAs or Kruskal-Wallis tests, as appropriate. Arousal was compared between final positive and negative sets to confirm matched emotional intensity. The final prime set comprised 5 positive and 5 negative age-relevant images matched in arousal and perceptual complexity.

### Phase 2: IA-AMP (Duration Optimization and Awareness)

#### Objective

The second phase aimed to test whether the IA-AMP can capture older adults’ implicit attitudes toward digital health technologies and to assess how variations in prime duration and awareness affect these responses.

#### Participants and Design

A total of 56 older adults participated across 2 sequential cohorts (Table 2). Batch 2A (n=29; mean age 65.0, SD 3.6 y; range 60‐71 y) used the initial awareness-probe interface, which prompted participants for 5 seconds to press the “6” key if they felt influenced by the preceding prime; otherwise, they were instructed to press nothing. Following preliminary testing in batch 2A and participant debriefing, the awareness interface was simplified in batch 2B (n=27; mean age 67.3, SD 4.9 y; range 61‐79 y) using a 4-second prompt requiring a space bar response. Batch 2A and batch 2B did not differ significantly in sex (*P*=.11) but differed modestly in age (*P*=.048) and substantially in education, with lower-education participants concentrated in batch 2A (*P*<.001).

The core AMP judgment task was unchanged across batches. Both batches used a 2 (prime valence: positive and negative) × 3 (prime duration: 75, 350, and 425 ms) within-participant design. Eligibility and ethics procedures mirrored phase 1.

**Table 2.** Demographic characteristics (study 2, n=56).

Variable	Batch 2A (n=29)	Batch 2B (n=27)	Total (n=56)
Age (y), mean (SD)	65.0 (3.6)	67.3 (4.9)	66.1 (4.4)
Age range (y)	60‐71	61‐79	60‐79
Sex, n (%)			
Female	21 (72.4)	14 (51.9)	35 (62.5)
Male	8 (27.6)	13 (48.1)	21 (37.5)
Residence, n (%)			
Urban	13 (44.8)	14 (51.9)	27 (48.2)
Rural	16 (55.2)	13 (48.1)	29 (51.8)
Education, n (%)			
Primary school or below	21 (72.4)	3 (11.1)	24 (42.9)
Middle school	8 (27.6)	17 (63.0)	25 (44.6)
High school or vocational	0 (0)	6 (22.2)	6 (10.7)
Undergraduate	0 (0)	1 (3.7)	1 (1.8)
Digital health technology user, n (%)			
Yes	22 (75.9)	22 (81.5)	44 (78.6)

#### Materials and Apparatus

Five positive and 5 negative images from phase 1 were used as primes. Neutral primes were not carried forward because they were not consistently rated as neutral during phase 1. Instead, a uniform gray square was used as a perceptual baseline. The targets were 40 abstract paintings [15], which were chosen because these images are evaluatively ambiguous [16]. All stimuli were shown in PsychoPy (version 2023.2.1; Open Science Tools Ltd) on a 24-inch monitor (500 × 400 pixels). Participants used a standard keyboard to record their responses, and each trial ended with a blank screen that stayed visible until a key was pressed.

### Procedure

#### Batch 2A

**Figure 2.**
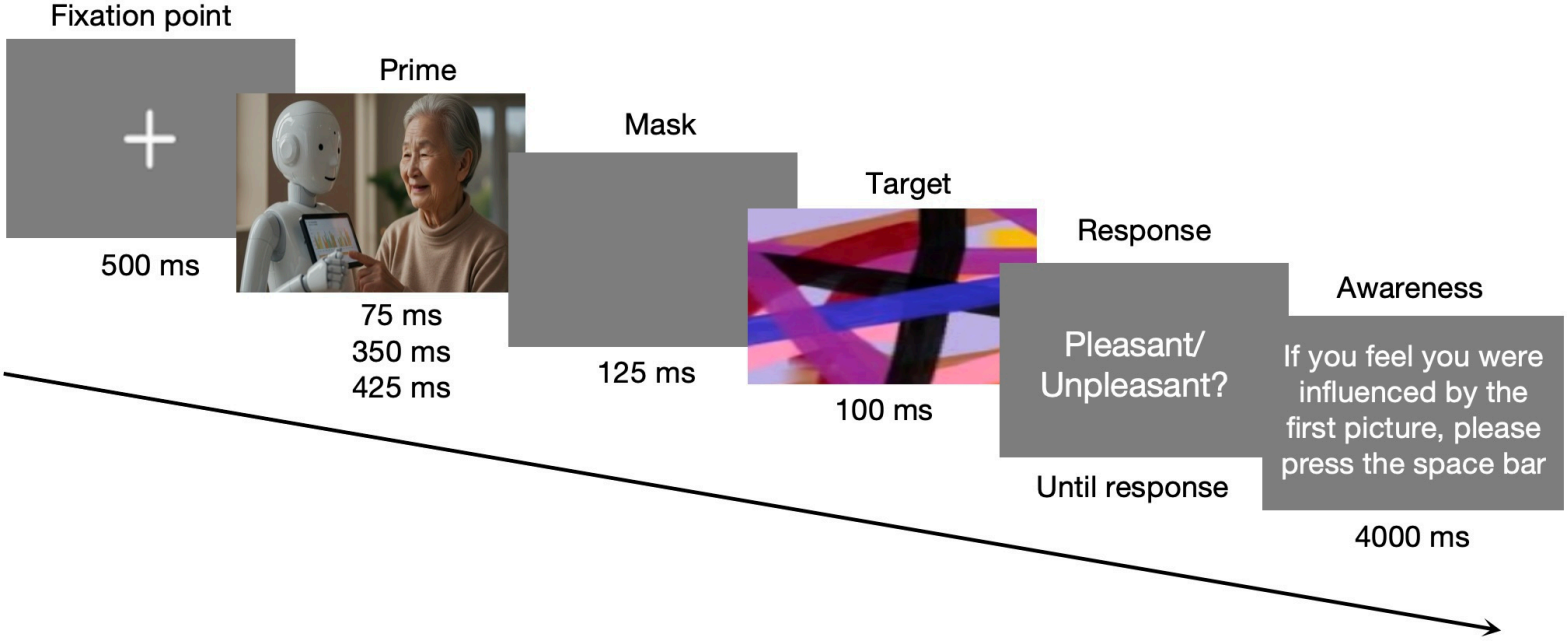
Trial structure of the influence-aware affect misattribution procedure used in batch 2B. Batch 2A followed the same sequence but included a 5-second awareness prompt requiring a “6” key press and displayed the awareness screen after all trials, including gray-prime trials.

Each participant first completed 10 practice trials to become familiar with the task, followed by 90 critical trials divided into 6 blocks of 15 trials each. The trials included positive, negative, and gray-square baseline trials, and prime duration (75, 350, or 425 ms) was randomized within each block. On every trial, a fixation cross appeared for 500 milliseconds, followed by the prime image for the assigned duration. After a 125-millisecond blank screen, a target abstract painting was shown for 425 milliseconds. A black-and-white mask then remained on the screen until the participant responded. Participants pressed the “E” key if they judged the painting to be less pleasant than average and the “I” key if it was more pleasant than average. Immediately after each target evaluation, an awareness prompt appeared for 5 seconds with the instruction to press the “6” key if they felt that the preceding image had influenced their judgment; otherwise, they waited for the next trial. A 1-second interval separated trials. Participants were free to rest between blocks for as long as needed and could press the space bar to continue when ready. Primes were sampled randomly without replacement, and the order of trials was fully randomized so that the task proceeded in a fixed forward direction. The absence of awareness responses under this initial interface prompted posttask debriefings, which informed a simplified awareness-probe interface for batch 2B (Figure 2). The core IA-AMP target judgment task was unchanged.

#### Posttask Probe

To supplement the trial-level awareness probe, 20 of the 29 participants were asked a brief set of follow-up questions immediately after completing the AMP task. These short probes were conducted in Mandarin by the researcher and focused on 4 domains: (1) task comprehension (eg, whether the instructions were clear), (2) interface usability (eg, ease of key presses and legibility of text), (3) cognitive load and fatigue (eg, whether the task felt confusing or tiring), and (4) perceptions of the awareness key (eg, whether participants understood what it meant).

Responses were grouped into 3 categories: (1) nonconscious processing, (2) procedural and interface-related barriers, and (3) motivational factors. These descriptive data were not analyzed as a full qualitative dataset but were used to contextualize the quantitative awareness results.

#### Batch 2B

Batch 2B used the same IA-AMP target judgment procedure as batch 2A, with a simplified awareness task based on batch 2A debriefings. Participants pressed the space bar instead of the “6” key to report awareness, and the awareness prompt appeared for 4 seconds. To reduce fatigue, the awareness screen was omitted during gray-square baseline trials. Therefore, awareness responses in batch 2B were collected only for positive-prime and negative-prime trials.

### Data Analysis

#### Outcome Measures and Data Handling

The primary inferential outcome was the trial-level target evaluation in the IA-AMP task, coded as 1 for “pleasant” and 0 for “unpleasant.” Consistent with standard AMP scoring, the AMP effect was summarized descriptively as the difference in the proportion of pleasant judgments following positive vs negative primes at each prime duration. “Pleasant judgments” refer to participants’ evaluations of the ambiguous target paintings, whereas “positive” and “negative” refer to the valence of the preceding prime image. These proportions are reported only to illustrate the magnitude of the effect; formal statistical inference was performed at the trial level, as described in the *Statistical Analysis* section.

The primary positive-negative AMP analysis included only trials with the validated set of 10 affective primes, comprising 5 positive and 5 negative images. Gray-square baseline trials were excluded from this comparison because they served as perceptual baseline trials and did not contribute to the positive vs negative effect. Trial logs were checked for missing or duplicate responses and for noncompliance (eg, identical responses across all trials), and no missing or duplicate responses or instances of noncompliance were detected.

The secondary outcome was trial-level awareness response frequency among batch 2B trials that included an awareness prompt, indicating whether participants reported that the preceding prime influenced their judgment on a given trial. Awareness analyses were restricted to batch 2B because the awareness-probe interface differed between batches, and gray-square baseline trials were not included because they did not receive awareness prompts. Descriptive feedback from posttask interviews in batch 2A was used to contextualize awareness-probe comprehension and interface-use issues but was not included in inferential analyses.

#### Statistical Analysis

The binary trial-level outcome described above was analyzed using binomial logistic generalized linear mixed-effects models (GLMMs). The primary AMP model was fitted to the final positive-prime and negative-prime trials and included prime valence, prime duration, their interaction, and batch as fixed effects, with random intercepts for participant and prime image. The focal prime valence × prime duration interaction was evaluated using likelihood-ratio comparisons between models with and without the interaction. Duration-specific positive vs negative contrasts were estimated at each prime duration, with Holm correction applied across the 3 contrasts. Model diagnostics were evaluated using checks for model convergence, overdispersion, and residual patterns.

Positive vs negative prime contrasts were estimated at each prime duration. Because contrasts were examined at all 3 durations, *P* values were adjusted using the Holm method. Odds ratios (ORs) with 95% CIs were reported. Descriptive AMP scores, calculated as positive minus negative differences in pleasant-response proportions, were reported to aid interpretation but were not used as the primary inferential test. To evaluate whether the findings were robust to additional stimulus-level control, an item-control sensitivity model was fitted with crossed random intercepts for participants, prime images, and target images.

Trial-level awareness responses were analyzed in batch 2B only because the awareness-probe interface differed across batches. Awareness was modeled as a binary outcome, indicating whether participants reported that the preceding prime influenced their judgment on a given trial. Gray-square baseline trials were excluded because they did not receive awareness prompts.

To evaluate whether AMP effects differed across the 2 sequential cohorts, batch-interaction sensitivity models were fitted with batch and its interactions with prime valence and prime duration as fixed effects, along with random intercepts for participant and prime image. A targeted likelihood-ratio test compared a model with all 2-way batch interactions to a model that additionally included the prime valence × prime duration × batch interaction. To assess whether differences in cohort composition accounted for the priming effect, the primary model was additionally refitted with participant age, sex, and education as covariates.

All analyses were conducted in R (version 4.5.3; R Foundation for Statistical Computing). Binomial logistic GLMMs were fitted using glmmTMB. Estimated marginal means, model-estimated probabilities, and duration-specific contrasts were obtained using emmeans, with Holm correction applied across the 3 contrasts. The focal prime valence × prime duration interaction was evaluated using a likelihood-ratio test comparing models with and without the interaction term. Model diagnostics were assessed using DHARMa.

## Results

### Phase 1: Stimulus Validation

#### Participant Characteristics

A total of 40 older adults (aged 60‐87 y; mean 68, SD 6.25 y; n=19, 47.5% female participants) completed the image-rating task. Two participants were excluded due to noncompliance (identical responses across trials). All remaining participants met the inclusion criteria and had normal or corrected vision.

#### Perceptual Feature Analysis

To evaluate whether the AI-generated images were perceptually balanced, we compared low-level visual properties (spatial frequency, chromatic complexity, and luminosity) across the 3 valence groups (Table 3).

**Table 3.** Means and SDs of perceptual features for AI-generated images by valence category (N=75).

Valence	Spatial frequency, mean (SD)	Color entropy, mean (SD)	Luminosity, mean (SD)
Positive	24.51 (1.95)	3.88 (0.19)	78.97 (9.13)
Neutral	23.71 (3.03)	3.84 (0.18)	81.55 (11.46)
Negative	22.52 (2.78)	3.80 (0.20)	85.28 (12.98)

**Figure 3.**
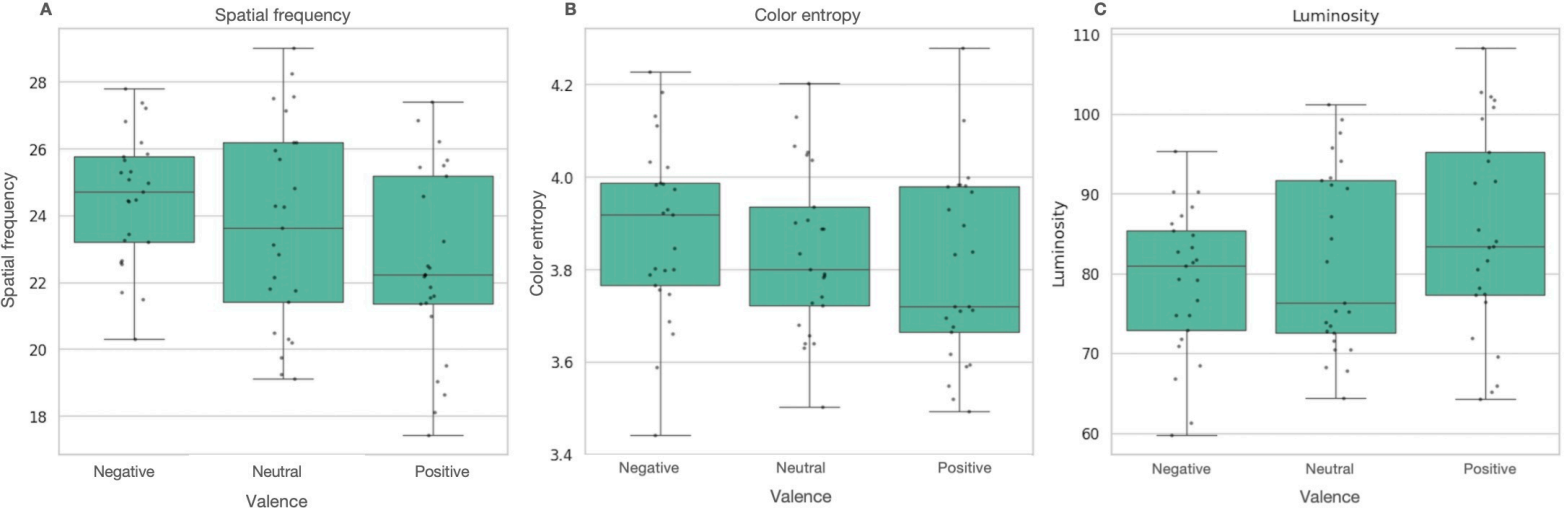
Box plots of perceptual features by valence category: (A) spatial frequency, (B) color entropy, and (C) luminosity.

Shapiro-Wilk tests were used to assess normality, and 1-way ANOVAs or Kruskal-Wallis *H* tests were applied as appropriate. Descriptive distributions of the 3 perceptual features across valence categories are presented in Figure 3. Shapiro-Wilk tests indicated that distributions were approximately normal for spatial frequency and color entropy across groups, but not for luminosity (neutral group *W*=0.913; *P*=.04). Accordingly, 1-way ANOVAs were used for spatial frequency and color entropy, and a Kruskal-Wallis test was used for luminosity. A significant effect of valence was observed for spatial frequency (*F*_2,72_=3.61; *P*=.03) driven primarily by differences between neutral and negative images. No significant differences were found for color entropy (*F*_2,72_=1.15; *P*=.32) or for luminosity (*H*_2_=2.95; *P*=.23). Because neutral images were not carried forward into the AMP task (phase 2), the spatial frequency effect was not considered relevant. Positive and negative image sets, which served as primes in phase 2, were comparable across all 3 perceptual dimensions.

#### Behavioral Ratings

##### Reliability of Ratings

Interrater reliability was assessed for valence, arousal, and relevance ratings using ICC(2,k) (2-way random effects model and absolute agreement [17]; Table 4). Because each participant rated either valence or arousal, but all rated relevance, per-image ICCs could not be estimated; instead, overall reliability estimates are reported. Results indicated excellent agreement for valence (ICC=0.99, 95% CI 0.99-0.99) and good agreement for relevance (ICC=0.81, 95% CI 0.75-0.87) and arousal (ICC=0.77, 95% CI 0.68-0.84; all *P*<.001; Table 2). Split-half correlations yielded similar results (valence *r*=0.986, arousal *r*=0.839, and relevance *r*=0.801), supporting the robustness of these estimates. Together, these findings demonstrate that ratings were highly consistent across participants.

**Table 4.** Intraclass correlation coefficients (ICC[2,k]) for interrater reliability of image ratings.

Dimension	ICC(2,k); 95% CI	*F* value (*df*)	*P* value
Valence	0.99; 0.99-0.99	105.96 (72, 1368)	<.001
Arousal	0.77; 0.68-0.84	5.99 (72, 1368)	<.001
Relevance	0.81; 0.75-0.87	7.12 (72, 2808)	<.001

##### Filtering and Categorization

To ensure that the stimulus set was thematically relevant and affectively unambiguous, images were filtered in several steps. First, we excluded images with mean relevance ratings below 4 on a 7-point scale. Second, images showing high variability across raters (SD≥1.5 for valence or relevance) were removed to eliminate ambiguous stimuli. Third, arousal ratings were standardized, and images outside the 95% CI (*z*-scores ±1.96) were excluded to reduce the influence of atypical extremes. Finally, participant ratings were compared with intended valence categories. Whereas positive and negative sets were retained, most neutral images were inconsistently classified; only 5 neutral images passed the criteria, which was deemed insufficient and unreliable. Consistent with affective norming practices (eg, IAPS [International Affective Picture System] and OASIS [Open Affective Standardized Image Set]) [18,19], neutral primes were therefore operationalized as perceptually simple gray squares in the subsequent AMP task.

From the filtered pool, 5 positive and 5 negative images were retained as primes for the AMP. All selected stimuli were rated as moderately arousing, with mean ratings falling within the 3.5 to 4.5 range. A Welch 2-tailed *t* test confirmed that mean arousal did not differ significantly between the positive (mean 4.15, SD 1.60; n=100) and negative (mean 4.17, SD 1.65; n=100) sets (*t*_198_=–0.09; *P*=.93). These results indicate that emotional intensity was successfully balanced across affective conditions.

### Phase 2: IA-AMP

#### AMP Effects

The primary positive-negative AMP analysis used the final validated affective-prime set of 5 positive and 5 negative images (gray-square baseline trials were excluded). Data were analyzed using a trial-level binomial GLMM that included prime valence, prime duration, their interaction, and batch as fixed effects, along with random intercepts for participant and prime image. The prime valence × prime duration interaction significantly improved model fit, as assessed by a likelihood-ratio test (*χ*^2^_2_=38.1; *P*<.001), indicating that the positive-negative AMP effect varied across durations.

**Figure 4.**
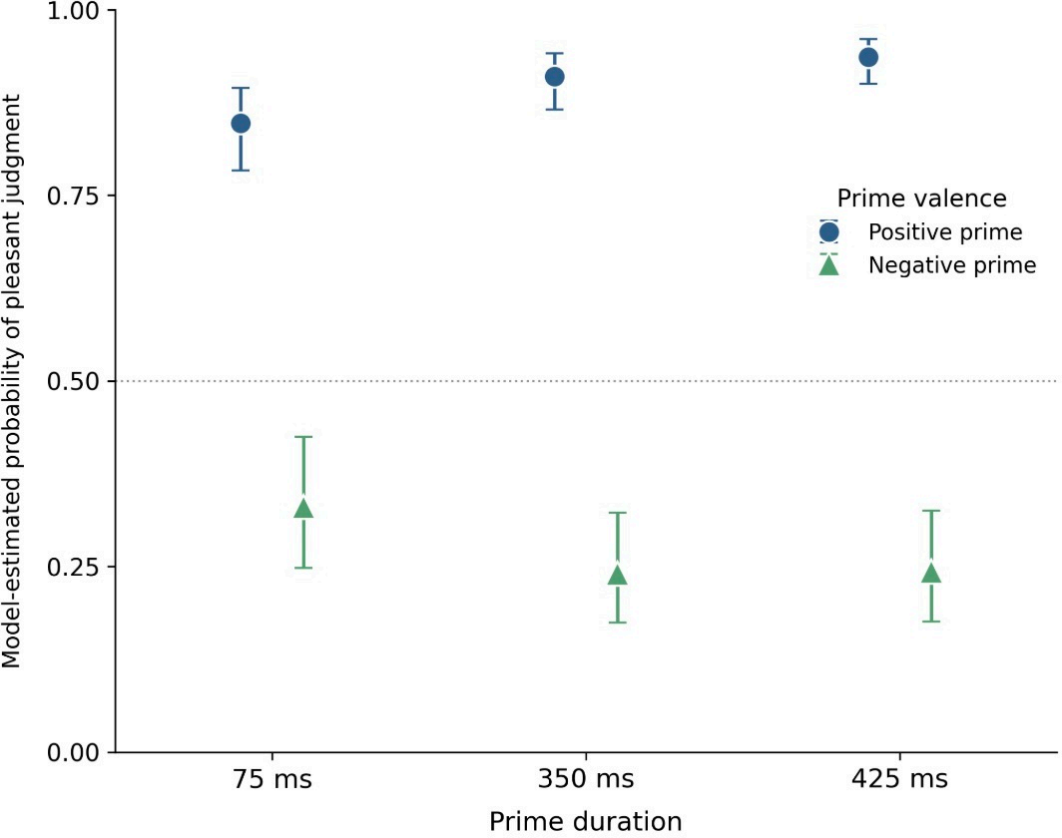
Model-estimated probability of a pleasant judgment by prime valence and prime duration. Circles represent estimated marginal probabilities for positive primes, and triangles represent negative primes, derived from the trial-level binomial generalized linear mixed-effects model (GLMM), averaged proportionally over batch, with 95% CIs back-transformed from the logit scale. The dotted line marks chance responding (0.5). Follow-up model-based contrasts showed that the positive-negative difference was smaller at 75 ms than at 350 and 425 ms (both Holm-adjusted *P*<.001), whereas the differences at 350 and 425 ms did not differ significantly (*P*=.17). Model-estimated probabilities differ slightly from the raw proportions in Table 5 because the GLMM adjusts for crossed random intercepts and is averaged proportionally over batch.

Descriptively, the raw AMP effect (the positive-negative gap in pleasant judgments) was smallest at 75 milliseconds (0.480) and increased to 0.625 at 350 milliseconds and 0.658 at 425 milliseconds. Full descriptive proportions and model-based estimates for each condition are reported in Table 5 and displayed in Figure 4.

**Table 5.** Proportion of pleasant judgments, raw affect misattribution procedure (AMP) effects, and model-based contrasts by prime valence and prime duration^a^.

Prime duration (ms)	Positive Pr(pleasant)	Negative Pr(pleasant)	Raw AMP effect	OR^b^ (95% CI)	*P* value
75	0.827	0.347	0.480	11.2 (5.8-21.6)	<.001
350	0.889	0.264	0.625	31.9 (16.0-63.4)	<.001
425	0.921	0.263	0.658	45.6 (22.1-94.0)	<.001

a Positive Pr(pleasant) and Negative Pr(pleasant) are pooled trial-level proportions. Raw AMP effect = Positive Pr(pleasant) − Negative Pr(pleasant). OR is the odds ratio for positive vs negative prime contrast at each duration, estimated from the primary trial-level binomial GLMM. *P* values are Holm-adjusted across the 3 duration-specific contrasts.

b OR: odds ratio.

Holm-corrected positive vs negative contrasts were significant at all durations: positive primes increased the odds of a pleasant judgment relative to negative primes by a factor of 11.2 at 75 milliseconds, 31.9 at 350 milliseconds, and 45.6 at 425 milliseconds (all *P*<.001; 95% CIs given in Table 5).

The magnitude of this effect differed by duration. The positive-negative contrast was significantly smaller at 75 milliseconds than at 350 milliseconds (log-odds difference=−1.046; *P*<.001) and at 425 milliseconds (log-odds difference=−1.404; *P*<.001). The contrast did not differ significantly between 350 and 425 milliseconds (log-odds difference=−0.359; *P*=.17). Affect transfer was, therefore, robust at all durations but weaker at 75 milliseconds than at the 2 longer durations, which were statistically comparable.

An item-control sensitivity model adding target-image random intercepts produced the same substantive fixed-effect pattern, with the prime valence × prime duration interaction remaining significant (*χ*^2^_2_=38.1; *P*<.001). The target-image SD was minimal (0.09 on the logit scale) relative to the participant and prime-image SDs (0.63 and 0.36, respectively), suggesting that the observed AMP effects were not primarily attributable to idiosyncratic features of particular target images.

#### Batch Sensitivity Analyses

Batch sensitivity analyses were conducted to evaluate whether AMP effects differed across the 2 sequential cohorts. A model allowing batch interactions improved fit relative to the primary model (*χ*^2^_5_=21.79; *P*<.001), indicating that batch moderated some aspects of the response pattern. Follow-up contrasts showed significant positive-negative priming at all durations in both batches. In batch 2A, positive vs negative ORs were 7.23 at 75 milliseconds, 25.19 at 350 milliseconds, and 31.86 at 425 milliseconds (all *P*<.001). In batch 2B, the corresponding ORs were 18.52, 43.32, and 72.82 (all *P*<.001).

However, the targeted prime valence × prime duration × batch interaction was not significant (*χ*^2^_2_=0.76; *P*=.68). This indicates that although batch 2B showed stronger overall positive-negative priming, the duration-dependent pattern of priming did not differ reliably between batches.

Because the 2 cohorts also differed in age and education (see Participants and Design section), we refitted the primary model adding participant age, sex, and education as covariates to assess whether these differences accounted for the priming effect. The prime valence × prime duration interaction remained significant (*χ*^2^_2_=38.0; *P*<.001), and none of the covariates significantly predicted pleasant judgments (for age *P*=.56, for sex *P*=.83, and for education *P*=.48). The duration-dependent priming pattern was therefore not attributable to differences in cohort composition.

#### Awareness Responses

No awareness responses were recorded in batch 2A under the initial awareness-probe interface. To clarify this zero-response pattern, 20 of the 29 batch 2A participants completed brief posttask probes in Mandarin. Responses were summarized descriptively and suggested that the absence of awareness responses reflected a combination of interface-related barriers, task-load issues, and conservative use of the awareness key, rather than definitive evidence of no prime awareness.

Awareness analyses were, therefore, restricted to batch 2B, where the simplified awareness interface was used. In batch 2B, reported influence awareness was common. Participants reported awareness of the preceding prime’s influence on 57.5% of prompted trials at 75 milliseconds, 66.2% at 350 milliseconds, and 68.3% at 425 milliseconds. A binomial mixed-effects model confirmed that awareness increased with prime duration: awareness was significantly lower at 75 milliseconds than at 350 milliseconds (*P*=.004) and 425 milliseconds (*P*<.001), with no significant difference between 350 and 425 milliseconds (*P*=.52). Participant-level summaries indicated that awareness reports were not limited to a small subset of participants. All 27 batch 2B participants reported at least 1 awareness event at each duration.

These findings indicate that the simplified batch 2B awareness-interface successfully elicited awareness reports, but they also show that reported influence awareness was frequent. Therefore, phase 2 should not be interpreted as showing that AMP effects occurred under minimal or absent awareness.

#### Awareness-Stratified AMP Effects

Exploratory awareness-stratified summaries showed that reported influence awareness substantially shaped AMP effects in batch 2B. On awareness-reported trials, target judgments were highly aligned with prime valence. Pleasant-response rates following positive primes were 92.0%, 94.0%, and 96.1% at 75, 350, and 425 milliseconds, respectively, whereas corresponding rates following negative primes were 4.5%, 3.0%, and 3.7%. This produced raw awareness-reported AMP effects of 0.875, 0.910, and 0.924, respectively.

On trials without reported awareness, positive-negative differences were much smaller. Pleasant-response rates following positive vs negative primes were 78.8% vs 77.3% at 75 milliseconds, 81.6% vs 73.5% at 350 milliseconds, and 85.6% vs 70.6% at 425 milliseconds, corresponding to raw AMP effects of 0.016, 0.081, and 0.150, respectively. These descriptive patterns indicate that the overall AMP effect was strongly associated with reported influence awareness (Table 6).

**Table 6.** Awareness-stratified pleasant-response proportions in batch 2B^a^.

Prime duration (ms)	Awareness status	Negative Pr(pleasant)	Positive Pr(pleasant)	Raw AMP^b^ effect
75	Aware	0.045	0.920	0.875
75	Unaware	0.773	0.788	0.016
350	Aware	0.030	0.940	0.910
350	Unaware	0.735	0.816	0.081
425	Aware	0.037	0.961	0.924
425	Unaware	0.706	0.856	0.150

a Proportions are descriptive trial-level values from batch 2B only (n=27). Awareness status reflects whether participants reported that the preceding prime influenced their judgment on a given trial. Raw AMP effect = Positive Pr(pleasant) − Negative Pr(pleasant).

b AMP: affect misattribution procedure.

Because awareness-reported trials showed near-complete separation, with highly consistent pleasant responses following positive primes and unpleasant responses following negative primes, ORs from awareness-stratified logistic models were extremely large and are not emphasized in the main text. The cell-level proportions provide a more interpretable summary of the awareness-stratified pattern.

#### Summary of Timing Pattern

Across both batches, the IA-AMP produced robust positive-negative affect-transfer effects. The effect was significantly smaller at 75 milliseconds than at 350 milliseconds and 425 milliseconds, whereas 350 milliseconds and 425 milliseconds did not differ significantly. Batch 2B showed stronger overall priming than batch 2A, but the duration-dependent pattern was consistent across batches.

The results did not identify a duration that simultaneously maximized affect transfer and minimized awareness. The 75-millisecond duration showed the lowest reported awareness but the weakest overall AMP effect. The 425-millisecond duration showed the largest descriptive AMP effect but also the highest reported awareness and the longest exposure duration. The 350-millisecond duration produced strong affect transfer comparable to 425 milliseconds while avoiding the longest exposure duration. Therefore, 350 milliseconds is best interpreted as a practical calibration point rather than a strictly low-awareness or fully implicit optimum. Reported influence awareness remained common and should be monitored and modeled in future older-adult AMP studies.

## Discussion

### Principal Findings

This study adapted and evaluated the IA-AMP to assess older adults’ affective responses toward digital health technologies. Phase 1 developed and validated age-relevant prime images depicting older adults in digital health contexts. The final positive and negative prime sets were emotionally distinct and comparable in arousal and low-level perceptual features, whereas neutral human images were not consistently rated as neutral and were, therefore, replaced by gray-square baseline stimuli in phase 2, consistent with prior AMP work [9].

Phase 2 evaluated AMP sensitivity across prime durations and examined the reported influence of awareness during affect transfer. Across 2 batches, participants were significantly more likely to judge ambiguous targets as pleasant following positive digital health primes than following negative primes. The magnitude of this effect was duration-dependent: the positive-negative contrast was significantly smaller at the standard 75 milliseconds duration and stronger at both longer durations, with no reliable difference between 350 and 425 milliseconds. This pattern suggests that very brief prime exposure may be insufficient for older adults, whereas moderately extended exposure improves affect transfer. This finding is consistent with broader evidence of age-related slowing in visual and cognitive processing speed [20,21], suggesting that standard AMP timing parameters developed for younger adults may require modification for older adults.

Awareness responses were not observed in the first batch but emerged after interface refinement in batch 2B. Posttask debriefings with batch 2A participants indicated that the absence of responses reflected interface comprehension barriers and conservative key-press behavior rather than a definitive absence of prime awareness. In contrast to the original expectation that awareness would remain relatively low, reported influence awareness in batch 2B was common, exceeding half of prompted trials at all durations. Awareness-stratified summaries further indicated that reported influence awareness substantially shaped the AMP effect, consistent with Hughes et al [13]. On awareness-reported trials, target judgments were highly aligned with prime valence, whereas positive-negative differences were much smaller on trials without reported awareness. These findings show that the IA-AMP can produce robust affect-transfer effects in older adults while also revealing that reported influence awareness plays an important role in those effects. Taken together, the results support 350 milliseconds as a practical timing point for older adults. It produced strong affect transfer comparable to 425 milliseconds while avoiding the longest exposure time, though it should not be interpreted as a strictly low-awareness or fully unconscious parameter.

The contrast between batch 2A and batch 2B is best interpreted as a methodological finding about awareness-probe design. The core IA-AMP target judgment task was unchanged across cohorts, and the awareness prompt appeared only after the pleasant or unpleasant target judgment had already been recorded. Batch-interaction sensitivity analyses showed that the duration-dependent priming pattern did not differ reliably between cohorts, supporting the robustness of the primary AMP finding. The zero-response pattern in batch 2A, therefore, limits awareness-related interpretation but does not compromise the primary AMP target-judgment outcome. Instead, it highlights that awareness probes for older adults must be designed with response accessibility, instruction clarity, and task burden in mind.

### Comparison With Prior Work

This work extends the AMP literature by situating the present findings at the intersection of cognitive aging, visual processing, and methodological debates about the role of awareness in AMP effects. First, in relation to cognitive aging, the study applies the AMP to older adults, a population that has rarely been the focus of AMP methodological development. Earlier AMP work showed that evaluative priming can emerge across relatively fast and slower presentation rates [14,22], but much of this evidence was generated in younger adult samples and nonhealth attitude domains. In the present study, the shortest duration produced the weakest positive-negative contrast, whereas the 2 longer durations produced stronger and statistically comparable effects. This pattern is compatible with the idea that older adults may require somewhat longer stimulus exposure for reliable perceptual registration and evaluative carryover [20,21], even when the basic AMP mechanism remains intact. The study also extends the ecological scope of the AMP by using age-relevant digital health imagery, rather than the racial, political, or general social attitude stimuli that dominate much earlier work [7], showing that ambiguous targets can be systematically biased by digital health primes in a later-life context.

Second, the awareness findings also place the present study within ongoing debates about the role of awareness in AMP effects across the broader implicit-cognition literature. Classic AMP accounts conceptualize the task as an indirect measure of automatically retrieved evaluative responses [9]. More recent work by Kurdi et al [14] suggests that awareness reports may sometimes be outcomes of AMP-consistent evaluative responding rather than causes of AMP effects, cautioning against treating reported awareness as straightforward evidence that awareness drove the AMP effect. In contrast, Hughes et al [13] argue that influence awareness can strongly moderate AMP effects, either by amplifying affect transfer through conscious re-evaluation or by qualifying the task’s status as a measure of implicit attitudes. The present findings do not resolve this broader theoretical dispute, but they show that, in an older adult digital health context, reported influence awareness materially shaped the interpretation of AMP scores. Overall, priming was robust, but reported influence awareness was frequent, and awareness-reported trials showed much stronger positive-negative differences than trials without reported awareness. Thus, the influence-aware design was not merely an added procedural feature; it was central to interpreting what the task measured. An additional consideration specific to this population is that older adults characteristically prioritize accuracy over speed and adopt more conservative response thresholds than younger adults [23]. This tendency may extend to awareness reporting such that older adults who detect any prime-related internal reaction are more inclined to endorse an explicit awareness probe when prompted, producing a conservative reporting bias that may have contributed to elevated trial-level awareness rates beyond what strict conscious prime recognition alone would predict. These findings suggest that IA-AMP effects in older adults should be interpreted as affect-transfer responses measured alongside awareness, rather than as straightforward evidence of awareness-free implicit attitudes or pure affect misattribution [13,24].

### Implications for Digital Health Research

The present findings have several implications for digital health research and evaluation with older adults when interpreted alongside adoption research showing that older adults’ technology use is shaped not only by perceived usefulness or ease of use [25,26], but also by emotional, social, relational, and contextual factors [27,28]. Prior work has consistently documented barriers to older adults’ digital health engagement, including limited digital literacy, physical and cognitive challenges, usability problems, privacy concerns, mistrust, and technology anxiety, alongside facilitators such as accessible design, provider endorsement, social support, and co-design [27-29]. Taken together, this body of work suggests that emotional and trust-related responses are not peripheral to digital health engagement—they are part of the adoption process itself.

The IA-AMP contributes a complementary measurement approach for studying affective barriers that conventional self-report methods may not fully capture, particularly given older adults’ susceptibility to social desirability bias and limited introspective access to automatic emotional reactions [4]. Innovation Resistance Theory helps situate what those affective barriers may represent by distinguishing functional barriers, such as usage, value, and risk, from psychological barriers, such as tradition and image, and by framing resistance as an active response rather than a passive failure to adopt [30]. In digital health contexts, older adults’ resistance may involve emotional discomfort, symbolic misalignment, fears about losing relational care, privacy and data-security concerns, distrust in diagnostic accuracy, and skepticism about care quality [31]. Low self-efficacy and technology anxiety may further create feedback loops that reinforce avoidance behaviors. The present findings are consistent with this perspective: digital health primes elicited rapid affective responses that biased target judgments, suggesting that affective and image-related components of resistance may be measurable at the trial level. Incorporating the IA-AMP into standard technology evaluation pipelines in community or clinical settings would allow developers to assess the “affective usability” of a digital health tool, its capacity to foster emotional comfort and trust, alongside traditional functional usability metrics.

### Limitations and Strengths

The primary strength of this work lies in demonstrating that IA-AMP can be adapted for older adults and digital health imagery with a relatively low technical burden. The study was intentionally designed with several simplifications to balance methodological control and participant accessibility. Participants completed the task on standard desktop computers under researcher supervision, suggesting that the procedure is feasible in clinical or applied research settings.

While the sample sizes were modest (phase 1: n=40; phase 2: n=56), they were appropriate for a feasibility study and consistent with prior AMP and implicit-attitude research [32]. Nonetheless, this sample limits generalizability and precludes the examination of subgroup differences (eg, by age or cultural background). Larger and more diverse cohorts will be needed to evaluate the stability of IA-AMP effects, establish psychometric reliability, and test predictive validity against real-world measures of technology adoption, which is necessary before clinical relevance can be established.

The sequential recruitment of the 2 cohorts introduces limitations that shape the interpretation of both the primary and awareness-specific findings. The original batch 2A awareness-interface yielded no recorded responses and was subsequently simplified for batch 2B. Furthermore, the cohorts differed in baseline characteristics, with lower-education participants and a modest age difference concentrated in batch 2A. Although adjusting for age, sex, and education indicated that these demographic differences did not account for the primary AMP effect, the potential influence of temporal context arising from sequential recruitment cannot be fully ruled out. Consequently, while the core duration-dependent priming pattern was robust across both batches, the awareness-related conclusions are tied to the simplified batch 2B interface. Finally, reported influence awareness was common in batch 2B, suggesting that IA-AMP effects in this context reflect affect-transfer responses measured alongside awareness rather than in its absence.

Taken together, the findings indicate that the IA-AMP is a feasible and scalable method for assessing older adults’ affective responses to digital health technologies. Its brevity, low verbal demand, and computerized format make it potentially useful as a complementary method in digital health evaluation, particularly when used alongside self-report, usability testing, and awareness assessment. However, its value should not be understood as providing a simple, awareness-free measure of hidden attitudes. Rather, the IA-AMP may be most useful as part of a multimethod evaluation framework that captures affect-transfer responses while explicitly monitoring reported influence awareness.

### Conclusions and Future Directions

The present work demonstrates the feasibility of using the IA-AMP to assess older adults’ affective responses to digital health technologies. A prime duration of approximately 350 milliseconds produced strong affect transfer comparable to 425 milliseconds while avoiding the longest exposure duration. These findings suggest that moderately extended prime durations provide a practical timing point for older-adult AMP studies, improving affect-transfer sensitivity relative to very brief exposures. The findings also demonstrate that reported influence awareness must be measured and modeled when using AMP paradigms with older adults. Future studies should expand on this foundation by recruiting larger and more diverse cohorts to evaluate the stability of IA-AMP effects, examining psychometric reliability and predictive validity in relation to real-world digital health engagement, and incorporating richer awareness measures to clarify the boundary between automatic and conscious affect transfer. The next phase of research should also integrate the IA-AMP into participatory design and implementation studies to test whether reducing implicit negativity or enhancing positive affect toward digital health technologies improves adoption and sustained use. Advancing this approach from feasibility to validated measurement will be critical for developing technologies that older adults not only can use but are also willing and affectively prepared to engage with in later life.

## Abbreviations

AMP: affect misattribution procedure
GLMM: generalized linear mixed-effects model
IA-AMP: influence-aware affect misattribution procedure
IAPS: International Affective Picture System
ICC: intraclass correlation coefficient
OASIS: Open Affective Standardized Image Set
OR: odds ratio
